# Management and Outcomes of Invasive Procedures in Individuals with Hemophilia A on Emicizumab Prophylaxis: A Single Center Experience

**DOI:** 10.3390/hematolrep15040062

**Published:** 2023-11-01

**Authors:** Karla Rener, Saša Anžej Doma, Martina Fink, Helena Podgornik, Irena Preložnik Zupan

**Affiliations:** 1 Department of Hematology, University Medical Center Ljubljana, Zaloška cesta 7, 1000 Ljubljana, Slovenia; karla.rener@mf.uni-li.si (K.R.); sasa.anzej.doma@kclj.si (S.A.D.); martina.fink@kclj.si (M.F.); helena.podgornik@kclj.si (H.P.); 2 Faculty of Medicine, University of Ljubljana, Vrazov trg 2, 1000 Ljubljana, Slovenia; 3 Faculty of Pharmacy, University of Ljubljana, Aškerčeva cesta 7, 1000 Ljubljana, Slovenia

**Keywords:** emicizumab, prophylaxis, hemophilia A, factor VIII, factor FVIIa, invasive procedures, perioperative care

## Abstract

Prophylactic treatment with emicizumab has become an important and effective bleeding prevention for people with hemophilia A (PwHA). Perioperative management of PwHA using emicizumab prophylaxis is still challenging due to a lack of experience. Medical records of perioperative management and outcomes were reviewed, and data were collected for adult PwHA receiving emicizumab and undergoing surgical procedures between August 2019 and July 2022 at the University Medical Center Ljubljana. Twelve surgical procedures were performed in eight PwHA (one with FVIII inhibitors) while on emicizumab prophylaxis. Three minor procedures included cataract surgery, cystoscopic lithotripsy, and percutaneous coronary intervention. Nine major surgeries included four osteosyntheses, necrectomy of chronic osteomyelitis with new ankle arthrodesis, two below-knee amputations, total knee replacement, and placement of ventriculostomy after a spontaneous intraventricular hemorrhage. No major bleeds, thrombotic events or deaths, or new inhibitors appeared. Our real-world experience demonstrates that minor and major surgeries can be performed safely in PwHA on emicizumab prophylaxis. Additional data are needed to optimize dosing/duration of additional hemostatic agents in diverse invasive procedures and complex clinical situations.

## 1. Introduction

Aging people with hemophilia A (PwHA) with a history of on-demand treatment often require surgical management of hemophilic arthropathy [1,2]. Additionally, increased life expectancy contributes to modern disease comorbidities, most commonly cardiovascular diseases, malignancies, diabetes, obesity, and dementia, all contributing to an increased risk of injuries and need for invasive procedures [1]. Notably, the increased risk of bleeding in hemophilia requires close perioperative monitoring [2].

Emicizumab is a bispecific antibody that mimics the function of factor (F) VIII and is approved for prophylactic treatment in PwHA with or without inhibitors [3]. The possibility of subcutaneous administration, long half-life, safety profile, and excellent treatment results with a significant reduction in annualized bleeding rates for all age groups has increased the use of emicizumab in many hemophilia treatment centers worldwide [4]. However, there are some important limitations of emicizumab, namely that it is not used for acute bleeding episodes, major surgeries, or invasive procedures. Recombinant activated FVII (rFVIIa) or FVIII replacement is additionally required in such cases, and laboratory monitoring of coagulation effects can be quite difficult [5,6,7,8,9,10]. Chromogenic FVIII activity assays with human reagents (CSA-H) allow the assessment of emicizumab activity. A chromogenic FVIII assay using bovine reagents (CSA-B) can be used to test the FVIII of any additionally administered FVIII concentrate or endogenous FVIII [2].

Surgical procedures were performed in PwHA receiving emicizumab prophylaxis in the HAVEN 1–4 [11] and STASEY trials [12]. However, as planned major surgeries presented exclusion criteria, performed surgeries were skewed towards minor interventions [11,12]. Real-world data on surgeries have also contributed to the evidence of safe emicizumab usage [13,14,15,16]; however, experience with invasive procedures for PwHA on emicizumab prophylaxis is limited, particularly in individuals without inhibitors.

The purpose of this manuscript is to report on our single-center clinical experience with all invasive procedures and peri-procedural hemostasis monitoring and management in adults with severe hemophilia A (HA) on emicizumab prophylaxis.

## 2. Materials and Methods

### 2.1. Ethical Statement

The University Medical Centre Ljubljana Ethics Commission approved the study (No. 140223/2) in concordance with the declaration of Helsinki. All patients included in this study provided informed consent.

### 2.2. Patients and Data Collection

Demographic and medical history data, the severity of HA, FVIII inhibitor status, and emicizumab dose/frequency were collected from the medical records of all adult PwHA on emicizumab prophylaxis undergoing surgical procedures between August 2019 and July 2022 at the University Medical Center Ljubljana, Slovenia.

Perioperative management data were also collected, including the type of surgical procedure, the factor concentrate used (preoperative dose and 14-day consumption), duration of hospital stay, laboratory monitoring parameters, procedure-related bleeding, and thrombotic complications. In two patients, we collected data on factor consumption for two surgical procedures performed before they started emicizumab prophylaxis.

### 2.3. Laboratory Analysis

For laboratory monitoring during and post-surgery/invasive procedures, CSA-H was used. CSA-B measurements were performed later on plasma samples of two patients stored at −80 °C, as previously described, to compare results with the CSA-H measurements [9]. Measurements were performed on ACL TOP 500 (Instrumentation Laboratory, Milan, Italy). Surgical procedures were classified as minor or major, as defined by Santagostino et al. (2015) [17].

### 2.4. Statistical Analysis

Data were retrospectively analyzed. Exploratory outcomes are reported using descriptive statistics. Statistical analysis was performed using MedCalc Statistical Software (version 19.6, MedCalc Software Ltd., Ostend, Belgium). Differences between the CSA results using human and bovine reagents for FVIII activity level were compared using Wilcoxon’s test and Spearman’s correlation coefficient (rho). A rho value > 0.75 was considered to be a strong correlation. A *p*-value < 0.05 was considered statistically significant.

## 3. Results

### 3.1. Patient Characteristics

In the cohort of male adult patients on emicizumab prophylaxis (N = 18), twelve surgeries were performed in eight patients (Table 1). The median age at the time of surgery was 53.5 years. All patients had severe HA, and one patient, who had two surgeries, had active FVIII inhibitors. The median duration of emicizumab exposure was 405 days. All patients received standard emicizumab prophylaxis of 3 mg/kg once every two weeks.

### 3.2. Management and Outcomes of Minor Invasive Procedures

Three minor interventions (two electives, one urgent) were carried out in three PwHA without inhibitors (Table 2). Two percutaneous interventions (PCI) with stent placement (classified as one procedure) were required urgently in one PwHA with an acute ST-elevation myocardial infarction (STEMI). In addition, cataract removal and cystoscopic lithotripsy were performed as elective procedures.

- PwHA case #1: Cataract procedure

Prophylaxis with a single standard half-life (SHL) rFVIII injection (20 IU/kg) was planned for PwHA case #1, aiming at an activity level above 0.40 IU/mL, as previously reported by Mauser-Bunschoten et al. (2013) [18]. Due to a coincidental blunt trauma of the elbow, patient received a preoperative rFVIII dose (50 IU/kg). FVIII activity levels were assessed by CSA-B and CSA-H (baseline = 0.01 and 0.21 IU/mL; 20 min after rFVIII administration = 0.78 and 1.25 IU/mL; and 24 h post-surgery = 0.23 and 0.63 IU/mL, respectively). No postoperative rFVIII dose was given.

- PwHA case #2: Cystoscopic lithotripsy

PwHA #2 received preoperative and prolonged postoperative SHL rFVIII due to hematuria. Consumption is presented in Table 2. Preoperative FVIII activity by CSA-H was 1.48 IU/mL and 0.75 IU/mL until postoperative day (POD) 10. On POD 15, the PwHA was readmitted for a short hospital stay due to urinary retention and relapse of hematuria and received an additional eight days of SHL rFVIII treatment (trough level of 0.70 IU/mL).

- PwHA case #3: Urgent percutaneous coronary interventions (PCI) after STEMI

SHL rFVIII injection (27 IU/kg) was administered before the PCI in addition to dual antiplatelet therapy (DAPT; ticagrelor (180 mg), acetylsalicylic acid (500 mg), and standard heparin). A drug-eluting stent (Resolute onyx 3.0 × 18) was inserted using radial approach due to subtotal stenosis of the left anterior descending artery (1st PCI). With the goal to maintain a FVIII level above 0.8 IU/mL (CSA-H), the patient received an additional dose of SHL rFVIII (17 IU/kg); chest pain and electrocardiogram (ECG) changes shortly after injection suggested a possibility of reinfarction. An urgent diagnostic coronary angiography (2nd PCI) was subsequently performed using a femoral approach and excluded in-stent thrombosis. Despite using a vascular closure device (AngioSeal^®^), the patient experienced mild local oozing from the puncture site. The patient received SHL rFVIII substitution until POD 5. To avoid higher peaks of FVIII, the daily amount of SHL rFVIII (25 IU/kg) was divided into three doses. Ticagrelor was replaced by clopidogrel on POD 2. During the first month of DAPT and regular emicizumab prophylaxis, four spontaneous joint bleeds required additional rFVIII applications. For this reason, emicizumab was replaced by regular prophylaxis with extended half-life (EHL) rFVIII (50 IU/kg every other day) with trough level 0.20 IU/mL (CSA-H). No additional spontaneous bleeding was reported subsequently on DAPT.

### 3.3. Management and Outcomes of Major Surgical Procedures

Nine major surgical procedures, five urgent and four elective, were carried out in five PwHA, one with inhibitors at the time of surgery (Table 2). Postoperative bleeds were reported for two urgent osteosynthesis interventions. Preoperative and the 14-day postoperative factor consumption is presented in Table 2. All major surgical interventions in PwHA without inhibitors on emicizumab prophylaxis were performed with rFVIII with an initial preoperative dose of 40–50 IU/kg, maintaining a trough level of 0.80 to 1.00 IU/mL for 3–5 days and lowering the intensity thereafter, in line with several guideline recommendations [6,7,8]. Since we guided our treatment according to the results of FVIII by CSA-H, VIII levels were maintained at the upper recommended levels. In the inhibitor patient, we used an initial preoperative dose of rFVIIa 90 μg/kg and tranexamic acid 1g and then dosed according to the clinical course. Thrombosis prevention involved non-pharmacological measures only. In all orthopedic and trauma surgeries, PwHA also received conventional tranexamic acid on the day of the procedure (1 g intravenous bolus preoperatively followed by 1 g continuous infusion for an initial 8 h).

- PwHA case #4 with inhibitors: Spiral fracture and surgery followed by osteosynthesis of pseudoarthrosis of the right femur

PwHA #4 suffered an accidental right femur fracture between the total knee and hip endoprosthesis just after the fourth loading dose of emicizumab. Internal fixation with a locking plate and lag screw was performed. Before surgery, rFVIIa (90 μg/kg) and tranexamic acid (1 g) were administered. The measured blood loss of 800 mL was within the expected range. rFVIIa dosing every 3–4 h until POD 4 resulted in a drop in the hemoglobin level and requirement for a red blood cell transfusion (2 units). Subsequently, rFVIIa dosing was every 6 h on POD 4 to 9, every 8 h on POD 9 to 13, and every 12 h on POD 13 to 15, which resulted in a stable hemoglobin level. Equivalent FVIII activity (CSA-H) measurements for emicizumab were from 0.06 to 0.11 IU/mL during the entire surgical procedure. On POD 15, rFVIIa was discontinued, and the PwHA was discharged the following day. The PwHA received a planned 5th dose (5th week) of emicizumab (3 mg/kg) on POD 2 and continued with a maintenance dose (3 mg/kg every two weeks) according to the treatment protocol. After eight months post-surgery, osteosynthesis of right femur pseudoarthrosis was necessary due to inadequate fracture healing. The PwHA received rFVIIa (90 μg/kg) preoperatively and two later doses at 3-h intervals. A slightly more intensive rFVIIa replacement (every 2–3 h in the first three days) with a higher 14-day consumption was administered, as presented in Table 2. There were no bleeding complications. Before starting emicizumab prophylaxis, this PwHA had two major orthopedic surgeries. The 14-day consumption of rFVIIa for these two operations was higher, namely 5.83 mg/kg (2014, a total left knee replacement) and 7.35 mg/kg (2017, a total right knee replacement).

- PwHA case #5: Complex re-arthrodesis of the left ankle

**Figure 1:**
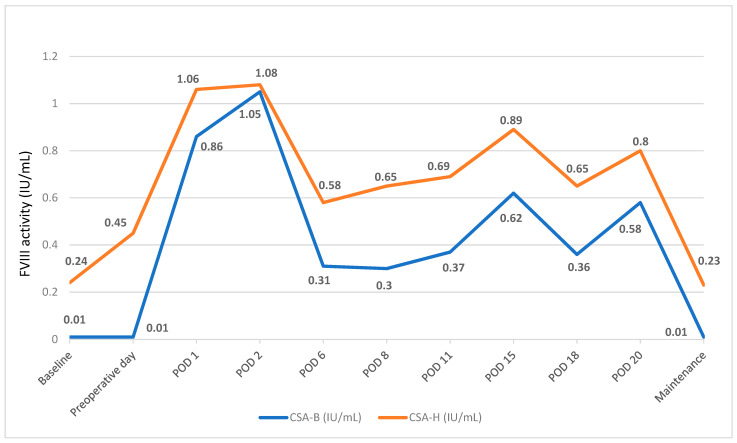
Differences between the CSA results using human and bovine reagents for FVIII activity level before, during and after left ankle re-arthrodesis.

This PwHA had a history of long-lasting chronic osteomyelitis with failure of multiple surgical and antibiotic treatment attempts, including a hyperbaric chamber treatment. Preoperative and 14-day consumption of rFVIII is presented in Table 2. The dosage was adjusted according to clinical course and CSA-H measurements. CSA-B measurements were subsequently performed from stored plasma samples (Figure 1). Correlation of FVIII measurements was excellent (Spearman coefficient: 0.972, *p* < 0.0001), with CSA-H measurement being 0.26 IU/mL higher (Bland–Altman). Below-knee amputation was needed ten months later due to inflammation persistence. FVIII activity was maintained above 0.50 IU/mL (CSA, H) and the PwHA was discharged on POD 14. For comparison purposes, the FVIII consumption for two additional surgical interventions performed before emicizumab prophylaxis, implant removal of osteosynthesis and necrectomy with revision of the skin graft, was 620 IU/kg and 655 IU/kg, respectively.

- PwHA case #6: Bilateral osteosynthesis of the tibia and fibula and below-knee amputation

This PwHA suffered severe polytrauma after a serious accident with multiple fractures of both legs and the 12th thoracic vertebra, which required urgent operative treatment. Bilateral osteosynthesis of both tibias was performed with damage-control surgery. Due to severe bleeding, a massive transfusion of blood products (red blood cells, fresh frozen plasma and platelets) was necessary. In addition, the PwHA was given preoperative EHL rFVIII (53 IU/kg), and additional EHL rFVIII (79 IU/kg) was required during surgery. CSA-H measurements were performed several times on the day of surgery and in the following days when vacuum-assisted closure replacement and daily necrectomies were needed. We maintained FVIII levels above 0.8 IU/mL. From POD 1 to 3, the patient received an EHL rFVIII (mean 65 IU/kg per day). On POD 3, a below-knee amputation of the left leg was performed due to severe tissue damage. FVIII activity above 0.8 IU/mL was maintained in the following days. The 14-day consumption of rFVIII is presented in Table 2.

- PwHA case #7: External ventricular drainage after intraventricular hemorrhage and osteosynthesis after hip fracture

After a year of successful prophylaxis with emicizumab, this PwHA without comorbidities was found unconscious. An urgent computerized tomography (CT) scan confirmed a spontaneous intraventricular hemorrhage. The patient received EHL rFVIII (53 IU/kg) in the emergency department and additional EHL rFVIII (80 IU/kg) during the insertion of an intraventricular catheter. The FVIII activity was maintained above 0.80 UI/mL (CSA-H). The 14-day consumption of rFVIII is presented in Table 2. CT scan controls showed gradual resorption of the hematoma. No vascular abnormalities, ischemic lesions, or signs of trauma were detected by two additional magnetic resonance imaging (MRI) scans. During prolonged, intensive FVIII replacement treatment, emicizumab prophylaxis was discontinued. The PwHA clinically improved and continued with rehabilitation, but then suffered a hip fracture on POD 30 after slipping off the edge of the bed due to balance instability and persistent cognitive impairment. FVIII level was maintained between 0.6 and 0.8 IU/mL (CSA-H); consequently, no significant bleeding developed. Femur osteosynthesis with *hip* screw and side plate was performed after an additional preoperative EHL rFVIII dose. The 14-day consumption of rFVIII is presented in Table 2. Only EHL rFVIII prophylaxis (21 IU/kg every other day) was continued to achieve a trough level above 0.20 IU/mL (CSA-H). The intensity of FVIII replacement was adjusted according to the PwHA’s actual needs, with higher levels administered during intensive physiotherapy.

- PwHA case #8: Total knee arthroplasty

Due to severe chronic arthropathy, an elective total knee arthroplasty was necessary for this PwHA. Consumption data are presented in Table 2. Treatment was complicated with sepsis; however, no hemorrhagic or thrombotic complication developed.

## 4. Discussion

Invasive procedures in PwHA on emicizumab prophylaxis remain a challenge for hemophilia treaters. Although the current treatment guidelines offer more practical information [5,6,7,8,9,10], clinical experiences are limited, especially for major surgeries in the non-inhibitor HA population. Managing invasive procedures in our diverse PwHA cohort adds important clinical information. We included all invasive procedures performed in PwHA on emicizumab prophylaxis over a period of almost three years.

No thrombotic events, thrombotic microangiopathy, or deaths occurred, and no new inhibitors to FVIII developed in our PwHA cohort undergoing invasive procedures. One minor and three major interventions were performed in an emergency setting. Notably, our cohort’s median age was higher than reported in the HAVEN 1, 3, and 4 trials of adult PwHA undergoing surgical procedures (53.5 vs. 31.5–46.6, respectively) [11]. Postoperative bleeding occurred in one of the three minor interventions (cystoscopic lithotripsy) and two of the nine major surgeries (two emergency osteosynthesis after severe injury, requiring blood transfusion), resulting in a postoperative no-bleeding rate of 75% in the whole patient cohort. A similar postoperative no-bleeding rate of 73% was reported in a review of medical records from PwHA on emicizumab prophylaxis at a single center in the United States [14]. As previously described, minor invasive procedures could be performed without additional hemostatic agents during emicizumab prophylaxis [11]. However, individual situations and disease differences require patient-specific decisions and treatment.

Two of our PwHA stopped emicizumab prophylaxis after an invasive procedure. One PwHA on DAPT after STEMI (case #3) reported spontaneous joint bleeds in the first month of treatment. The coronary pathology and the type of stent the PwHA received required 1-year DAPT. The World Federation of Hemophilia (WFH) guidelines suggest FVIII trough levels ≥ 0.15–0.3 IU/mL for PwHA during DAPT [2]. Modified European Society of Cardiology (ESC) guidelines for PwHA without inhibitors on emicizumab prophylaxis concluded with a 70% expert consensus that there is no need for additional factor replacement during DAPT [19]. Nagao and co-workers reported successful two-months bleeding protection with emicizumab during DAPT in a PwHA; however, administration of rFVIII every other day to maintain a FVIII trough of 0.15–0.3 IU/mL was deemed too burdensome [20]. In our PwHA cohort, equivalent activity of FVIII (CSA-H) while on emicizumab was between 9% and 11%. An open question remains whether the dose of emicizumab could have been increased to prevent spontaneous bleeds in PwHA rather than switching them to rFVIII prophylaxis [21]. However, this PwHA completed one year of DAPT and continues on rFVIII prophylaxis. We discontinued emicizumab in another PwHA (case #7) with intraventricular hemorrhage in whom the etiology of bleeding was not explained. Even though this PwHA had no bleeding for a year on emicizumab, we decided on a more intensive prophylaxis to maintain a FVIII trough level above 0.2 IU/mL (CSA-H) for six months after intracranial bleeding. Notably, however, intracranial bleeds can occur with any prophylaxis therapy.

As an exploratory analysis, we compared the 14-day consumption of rFVIII for similar orthopedic surgeries in the same patient (case #5) performed before and on emicizumab prophylaxis. Importantly, we identified a 40% lower perioperative consumption of rFVIII on emicizumab prophylaxis. In the PwHA with inhibitors (case #4), we also observed a trend of lower rFVIIa use (9%) for an orthopedic procedure with concomitant use of emicizumab. The hospital stay was shortened for this PwHA on emicizumab therapy since rFVII was not needed for more than two weeks. Due to differences in the surgery type, it was not possible to estimate the precise factor consumption, as reported in the literature [22]. A comparison of the perioperative management of the PwHA with inhibitors undergoing total knee replacement before and after initiation of emicizumab showed a 45% reduction in rFVIIa usage during the second procedure [22].

In case #6, the extent of the injury in the case of polytrauma resulted in a complex hemostatic disorder with persistent bleeding during the first three days despite intensive replacement of FVIII and other clotting components in the form of fibrinogen, fresh frozen plasma, platelets, and red blood cells. Regular emicizumab prophylaxis was continued, although there was a loss of emicizumab due to bleeding. Regarding the hypothesis that FVIII at a higher dose displaces emicizumab from binding sites on FIX and X, it is clear that during the intensive phase of treatment in this PwHA, it had little value. Moreover, the question remains about PwHA continuing emizicumab treatment while receiving prolonged intensive FVIII replacement therapy. PwHA case #6 did not develop any thrombotic complications despite intensive substitution and extensive tissue damage.

Interference of emicizumab with available laboratory coagulation tests poses a particular challenge in daily clinical practice. Unfortunately, there was no opportunity to measure CSA-B at the time of the surgeries, so we relied on CSA-H and extensive previous clinical experience. With the knowledge that concomitant use of emicizumab and rFVIII concentrates results in displacement of emicizumab from active sites in the coagulation cascade, we expected only the action of added FVIII in practice [23]. For additional insight into the pharmacokinetics of FVIII, we performed retrospective CSA-B measurements from frozen samples. Results of FVIII activity were compared in some patients, as exemplified in Figure 1 for PwHA case #5. At higher activities of FVIII, we observed smaller differences between the CSA-H and CSA-B results. Similar findings were reported in the literature of a PwHA without inhibitors who underwent total arthroplasty of the right elbow [24]. Although FVIII was dosed based on measurements of FVIII activity by CSA-H instead of the recommended CSA-B, there were only three perioperative bleeds (two major bleeds in the context of severe trauma and one minor bleed after bladder lithotripsy). Our experience has led us to believe that the availability of appropriate laboratory assays is important but not crucial for appropriate patient management.

In summary, despite being a small patient group, this real-world patient cohort contributes further to the results from clinical trials and the growing body of real-world evidence, demonstrating the safe management of invasive procedures in PwHA receiving emicizumab prophylaxis, regardless of FVIII inhibitor status, age, and other risk factors. However, questions remain for individual patients about the sufficiency of prophylactic treatment with emicizumab. Additional experience from real-world and research will undoubtedly contribute to better management of PwHA, requiring more complex treatments for comorbidities and invasive procedures in the future.

## Figures and Tables

**Table 1:** Basic characteristics for all surgical cases.

Characteristics	N = 12 ^†^
Age at the time of surgery, years	
Median (range)	53.5 (32–66)
History of inhibitors, n (%) ^‡^	2 (16.7)
Surgical classification, n (%)	
Major	9 (75.0)
Minor	3 (25.0)
Type of procedure, n (%)	
Orthopedic	8 (66.7)
Other	4 (33.3)
Bleeding episode, n (%)	
Yes	3 (25.0)
No	9 (75.0)
Duration of emicizumab exposure, days	
Median (range)	405 (26–608)

^†^ Twelve surgical cases are reported for eight patients; ^‡^ Inhibitors present in one patient undergoing two surgical procedures.

**Table 2:** Summary of management and outcomes of minor and major surgical interventions in PwHA on emicizumab prophylaxis.

PwHA Case #	Age at Time of Surgery, Years	Inhibitor Status (Yes/No)	Procedure/Surgery Type (Elective/Urgent)	Time on EMI, Days ^†^	Preoperative Factor Dose rFVIIa (µg/kg) rFVIII (IU/kg)	Factor Consumption (Days)	Hospitalization (Days)	Bleeding Episode * (Yes/No)	Overall Outcome
Minor surgical procedures									
1	55	No	Cataract removal (elective)	308	70 IU/kg	70 IU/kg (1 day)	0	No	Visual acuity improved.
2	66	No	Cystoscopic lithotripsy (elective)	230	50 IU/kg	285 IU/kg (10 days)	3	Yes	Bladder stones were successfully removed.
3	32	No	Percutaneous coronary intervention with stent placement (urgent) ^‡^	362	27 IU/kg (1st PCI); 17 IU/kg (2nd PCI)	130 IU/kg (4 days)	6	No	Switching to FVIII prophylaxis while on DAPT.
Major surgical procedures									
4	50	Yes	Osteosynthesis after spiral femur fracture (urgent)	26 (end of induction)	94 µg/kg	5.08 mg/kg (14 days)	18	Yes	Successful osteosynthesis.
	51	Yes	Osteosynthesis of pseudoarthrosis (elective)	279	90 µg/kg	6.54 mg/kg (14 days)	13	No	
5	56	No	Necrectomy of chronic osteomyelitis with re-arthrodesis (elective)	274	40 IU/kg	386 IU/kg (14 days)	22	No	Walks with the help of a leg prosthesis.
	56	No	Below-knee amputation (elective)	608	37 IU/kg	381 IU/kg (14 days)	15	No	
6	56	No	Bilateral osteosynthesis of the tibia and fibula (urgent)	596	53 IU/kg	506 IU/kg (14 days)	36 (one hospitalization)	Yes	Survived, remained disabled.
	56	No	Below-knee amputation (urgent)	599	26 IU/kg			No	
7	51	No	External ventricular drainage after intraventricular hemorrhage (urgent)	448	53 IU/kg	320 IU/kg (14 days)	58 (one hospitalization)	No	Physical and cognitive improvement. Successful osteosynthesis. Switch to FVIII prophylaxis.
	51	No	Osteosynthesis after hip fracture (urgent)	478	27 IU/kg	253 IU/kg (14 days)		No	
8	52	No	Total right knee arthroplasty (elective)	567	50 IU/kg	442 IU/kg (14 days)	16	No	Better mobility, less pain.

^†^ All PwHA were on emicizumab (3 mg/kg) prophylaxis once every two weeks. ^‡^ PwHA experienced a STEMI after one year on emicizumab. * Bleeding during hospitalization. EMI, emicizumab; IU, international unit; rFVIII, recombinant factor VIII; rFVIIa, recombinant activated factor FVII; PCI, percutaneous coronary intervention; PwHA, person with hemophilia A; STEMI, ST segment elevation myocardial infarction.

## Data Availability

The data presented in this study are available on request from the corresponding author. The data are not publicly available due to privacy.
